# One Size Doesn’t Fit All: An Exploratory Typological Approach to Understanding Criminal Career Heterogeneity in Intimate Partner Homicide

**DOI:** 10.1177/00938548241257604

**Published:** 2024-06-25

**Authors:** Olivier Péloquin, Julien Chopin, Francis Fortin, Jean-Pierre Guay, Eric Chartrand, Sarah Paquette

**Affiliations:** University of Montreal; University of Lausanne; Simon Fraser University; Laval University; University of Montreal; Sûreté du Québec; University of Portsmouth

**Keywords:** intimate partner homicide, criminal career, latent profile analysis, heterogeneity, trajectories

## Abstract

Approximately one in seven homicides globally is committed by a partner within an intimate relationship. While criminology research on intimate partner homicide (IPH) perpetrators is extensive, their interactions with law enforcement remain underexplored. This study examines the criminal trajectories of IPH perpetrators to ascertain whether they exhibit common or diverse patterns. Utilizing data from Quebec’s official criminal events database, the study analyzes variables concerning the criminal histories of 1,780 individuals involved in attempted or completed IPH through latent profile analysis. Findings indicate five distinct profiles among IPH perpetrators: one-time, low-volume intimate partner violence (IPV), moderate-volume IPV, high-volume violence, and high-volume polymorphous perpetrators. The external validity of these profiles is assessed using additional criminal career, contextual, and situational variables. Implications for the justice system’s practices and challenges are also discussed.

## Introduction

In 2018, an intimate partner was found responsible for one out of every five solved homicides in Canada (Roy & Marcellus, 2019). In the same year, police organizations reported 1,060 homicides or attempted homicides between intimate partners (Conroy et al., 2019). Homicide is the most severe crime in the Canadian Criminal Code and more specifically, intimate partner homicide (IPH), can be associated with the suicide of the perpetrator as well as the deaths of children or other family members. Any homicide poses a significant cost to society: In 2014, homicide cost the Canadian Justice System, on average, US$371,514.25 per case (Gabor, 2015). Despite solid groundwork by researchers, much work remains to be done to better understand IPH perpetrators. Only a few studies have used a typological approach to consider heterogeneity in the criminal careers of individuals who commit IPH (Dixon et al., 2008; Kivisto, 2015; Vignola-Lévesque & Léveillée, 2021). These previous studies, while suggesting that there are different categories of IPH perpetrators, are limited by the nature of the data used: Researchers were mainly interested in the psychological aspects of IPH perpetrators, and their samples were therefore drawn from forensic psychiatry. Beyond the obvious problem of sample size, these studies examined only IPH perpetrators who presented with mental disorders and required psychological intervention. This limitation has created a significant knowledge gap for police attempting to understand IPH perpetrators with a criminal trajectory lens, as no previous study has looked at IPH perpetrators in terms of other criminal activities. The study of criminal careers, an important area of criminological research, makes it possible to better understand and predict criminal recidivism (e.g., Gottfredson & Hirschi, 1990). A wealth of evidence from studies using representative official data of the criminal careers of those involved in intimate partner violence (intimate partner violence perpetrators) strongly shows that incidences of physical violence involving a partner often occur more than once, rather than one-off, (Hilton & Eke, 2016; Tanskanen & Aaltonen, 2022) and that two-thirds of IPVO had a criminal career prior to committing their first act of intimate partner violence (Hilton & Eke, 2016).

The issue of IPH has received very little attention and, while general trends suggest that perpetrators who kill their partners have longer criminal careers than perpetrators whose attacks do not lead to death (Campbell et al., 2017; Dobash et al., 2004; Eriksson et al., 2019), determining the validity of this view requires a better understanding of how the criminal careers of those involved in IPH are organized and articulated. Also, most of the time, official judicial system data is the sole known reference point for an individual prior to intervention by law enforcement agencies, who often serve as a first-line response in many cases. Hence, it is crucial to endeavor to discern the diverse criminal career trajectories that culminate in IPH because it helps identify intervention points and risk factors across an individual’s life that could lead to IPH. Understanding these trajectories allows for the development of specific prevention strategies and contributes to a deeper theoretical understanding of IPH by considering the longitudinal interplay of life experiences and criminal behavior. This approach not only aids in crafting evidence-based interventions but also enhances our theoretical models, providing a comprehensive view of the factors contributing to IPH. Ultimately, this perspective is vital for developing proactive and holistic approaches to prevent intimate partner violence and homicide. Consequently, this study examines the criminal career patterns of individuals involved in IPH (both attempted and completed) to determine if the paths associated with IPH are homogeneous or heterogeneous.

### The Criminal Career Paradigm

The criminal career paradigm, a framework for documenting and studying criminal trajectories, makes it possible to understand criminal behavior by looking at a longitudinal sequence of criminal acts committed by an individual during a given period (Blumstein et al., 1986). The number of studies using this paradigm has been increasing since the 1970s, in part, because the framework has contributed to the development of new theories and approaches to understanding crime (e.g., Life course theory; see Sampson & Laub, 2015). Determining the part crime plays in an individual’s life is a complex operation and has increasingly led to the use of innovative and more complex types of analysis (see longitudinal analysis and group-based modeling; Sullivan & Piquero, 2016). According to Blumstein et al. (1986), a criminal career can be understood in terms of four core dimensions: participation, frequency, seriousness, and duration. The participation dimension is defined as participation in crime over a lifetime and can be provided in several ways, for instance, either as an aggregate or percentage of the number of individuals who have committed a certain crime or as a record of an individual’s participation in various categories of crime (Blumstein et al., 1986). The concept of participation has given rise to another important idea in the criminal career framework — specialist versus generalist (i.e., are individuals who commit crimes highly specialized in one type of crime or do they commit various types of crimes?). The second dimension, frequency, refers to the number of crimes a person commits during a given period and is an important measure that can be used to assess the scale of an individual’s criminal career (Blumstein et al., 1986). The third dimension, seriousness, assesses the severity of a perpetrated offense. Calculating seriousness often involves the use of pre-made indexes, such as the Statistic Canada Seriousness of Crime Index, which is based on the perceived gravity of a crime as well as its cost to society (see Statistics Canada, 2021). Finally, the duration dimension provides a framework for analyzing an individual’s persistence and desistance in crime over the duration of a criminal career (Sullivan & Piquero, 2016).

### The Criminal Career of IPH Perpetrators

Few previous studies of IPH have used the criminal career perspective, focusing instead on the characteristics of IPH perpetrators’ crimes and suggesting that they can be divided into three categories: general level of participation in crime, participation in IPV-specific crimes, and categories or range of crimes committed (Eriksson et al., 2019). Studies highlight that most IPH perpetrators have a criminal history (Dobash et al., 2004; Eriksson et al., 2019; Thomas et al., 2011). Eriksson et al. (2019), using a sample of 68 individuals involved in IPH in Australia, found that 89.2% had committed at least one criminal offense prior to the homicide. Their findings agree with those of Dobash et al. (2004), who found that 72.6% of the 106 IPH perpetrators in their sample had a prior criminal conviction, and Thomas et al. (2011), who report that 92.4% of the 71 IPH perpetrators they studied had a prior arrest. It is interesting to put these results in contrast with studies on all types of homicide perpetrators, a cohort study in Illinois found that nearly 30% had no prior arrest (Cook et al., 2005), while a study on repeat homicide perpetrators found that 40% had no prior arrest (Delisi & Scherer, 2006). Empirical evidence that IPH perpetrators have participated in IPV-specific offenses is less consistent. In a review focused on individuals involved in IPH, Kivisto (2015) found that the percentages of those with a history of IPV varied widely, ranging from 21.7% to 76.5%, although this might be explained by the different data sources used in these studies (e.g., self-report vs. police data). Belfrage and Rying (2004), who used official police data, found that 36% of IPH perpetrators had been involved in other IPV events in the past. With regard to the participation of individuals involved in IPH in other types of crime, studies have shown that their criminal careers were multifaceted (Eriksson et al., 2019; Thomas et al., 2011). Eriksson et al. (2019) examined crime variety on a seven-item scale (0–7) and found that, on average, IPH perpetrators had a score of 2.8 (Eriksson et al., 2019). They used a three-level Likert-type scale to investigate the frequency of IPH perpetrators participation in crime in general, asking participants to self-report how frequently they had committed crimes. The results showed that 58.3% were in the no/low-frequency tier, 30% in the medium-frequency tier, and 11.7% in the high-frequency tier (Eriksson et al., 2019). Dobash et al. (2009) studied IPH perpetrators and found that 16.9% had their first contact with the criminal justice system early in life (13 years of age), while Eriksson et al. (2023) found that 32.8% of the IPH perpetrators in their sample had committed a least one criminal offense before adolescence (13 years of age).

### IPH Perpetrators and IPH Event Characteristics

Regarding the characteristics of IPH perpetrators and events, previous study results were inconsistent. Most agreed that IPH perpetrators were male and middle-aged (around the age of 40 at the time of the incident; Eke et al., 2011; Elisha et al., 2010; Kivivuori & Lehti, 2012; Loinaz et al., 2018). However, the analysis of their employment and relationship status produced conflicting results. Kivivuori and Lehti (2012) found that 57% of IPH perpetrators were unemployed, while Eke et al. (2011) reported that approximately one-third (37%) of IPH perpetrators did not have a legal occupation at the time of the IPH event. As for relationship status, studies suggested that IPH could occur both during and after the end of an intimate relationship, but the prevalence of active relationships at the time of an IPH event remained unclear (Eke et al., 2011; Thomas et al., 2011). Results regarding the context surrounding the homicide also varied. The role of alcohol intoxication during IPH events was uncertain, as some studies reported a high prevalence (Kivivuori & Lehti, 2012), while others found a low prevalence (Dobash et al., 2009). Interestingly, a recent study by Overstreet et al. (2021) found that alcohol/drug use was less prevalent in IPH cases than in IPV cases and suggested that this could be due to intoxication weakening and disorienting perpetrators, making a lethal outcome less likely. Several studies emphasized that the presence of firearms was a strong predictor of lethal outcomes in IPV (Campbell et al., 2017; Cooper & Smith, 2011; Overstreet et al., 2021; Spencer & Stith, 2020; Thomas et al., 2011).

### Heterogeneity and IPH Perpetrators

While most previous research provided a general overview of IPH perpetrators and IPH event characteristics, some researchers looked at IPH perpetrators heterogeneity and reported differences between IPH perpetrators (Dixon et al., 2008; Dobash & Dobash, 2016; Vignola-Lévesque & Léveillée, 2021). The heterogeneity hypothesis was tested with psychological (e.g., low self-control) and criminal career indicators (e.g., history of IPV; Kivisto, 2015). Interestingly, findings converged, suggesting that there are three or four different groups of IPH perpetrators (Dixon et al., 2008; Kivisto, 2015; Vignola-Lévesque & Léveillée, 2021). Dixon et al. (2008) found three subtypes in a sample of 72 IPH perpetrators: low criminality/low psychopathology (15%), moderate-high criminality/high psychopathology (36%), and high criminality/low-moderate psychopathology (49%). In a review of the existing literature, Kivisto (2015) identified four subgroups: mentally ill (no IPV history), undercontrolled/dysregulated (episodic IPV history), chronic batterer (persistent IPV history), and overcontrolled/catathymic (no IPV history). Vignola-Lévesque and Léveillée (2021), in a sample of 67 individuals involved in IPV perpetrators, found three subgroups of IPH perpetrators in four clusters of IPVOs: the homicidal abandoned partner (19.4%), the controlling violent partner (34.3%), and the unstable dependent partner (22.4%). Findings from these studies suggest that IPH perpetrators are a highly heterogeneous group. This latter finding may be the reflection of the diversity of the IPV perpetrators population. As a matter of fact, most studies about this subject among IPV perpetrators found three to four similar profiles (Holtzworth-Munroe & Stuart, 1994; Peters et al., 2022). For example, Peters et al. (2022) found three subgroups of IPV-offending man: IPV-Specific (30%), generally violent/antisocial (20%), and IPV and generally violent/antisocial (50%).

A review of the literature showed that there is little research on the criminal careers of IPH perpetrators and that, while the few studies have played an essential role in exploring and understanding the topic, they have several shortcomings. First, the notion of career criminality is considered only as a taxonomic concept, that is, the presence or not of prior violence, ignoring the complexity and nuances available in the criminal career perspective. Diving into these complexities is essential, as it allows us to differentiate between individuals who at first glance may seem similar on the surface. Second, most studies have small sample sizes, which leads to significant variability in the results. Third, most studies take a psychological approach to the study of IPH perpetrators, whereas all these factors, while interesting, are not readily available to frontline workers. This is where the criminal career shines, being simple to understand and readily available to police officers. We believe that a more detailed analysis of IPH perpetrator criminal careers based on a substantial sample could lead to both theoretical and practical advances.

## Aim of Study

The literature review revealed that the research on IPH perpetrators’ criminal careers remains limited and scarce. While the existing studies have played an essential role in exploring and understanding the topic, they exhibit several shortcomings. First, the concept of career criminality is examined solely as a taxonomic notion, indicating the presence or absence of prior violence, disregarding the complexity and nuances inherent in the criminal career perspective. Delving into these complexities is crucial as it allows differentiation between individuals who may initially appear similar on the surface. Second, most studies have small sample sizes, resulting in significant variability in the outcomes. Third, the majority of studies have adopted a psychological approach to studying IPH perpetrators. To augment the existing knowledge base and enhance the understanding of the phenomenon, we contend that it is crucial to examine it through various theoretical and explanatory lenses, especially the perspective of criminal careers, as employed in this study. We contend that a more comprehensive analysis of IPH perpetrator criminal careers, based on a substantial sample, could pave the way for both theoretical and practical advancements. Therefore, in this study, we examine the heterogeneity of the criminal histories of IPH perpetrators to determine if some trajectories can be associated with different dimensions of the criminal career framework or with specific crime characteristics. As the first to investigate this question, the study is exploratory by nature; thus, two hypotheses emerged from these goals:

**Hypothesis 1 (H1):** IPH perpetrators’ follow heterogeneous criminal career trajectories
**Hypothesis 2 (H2):** IPH perpetrators’ criminal career trajectories are associated with identifiable crime characteristics

## Method

### Sample

Data used in this study were taken from an official Quebec police database that includes all criminal events recorded in the province of Quebec in Canada. This database is known as Index général du Module d’information policière (Index général/MIP). This system, an integral part of the CRPQ, was set up in 1980 to increase the efficiency of information retrieval to facilitate crime-solving. The presence of crimes recorded on the computer system before its implementation (read before the early 1980s) can be explained by the fact that these investigations, initially unsolved, were reactivated after the computer system was implemented due to new evidence or the exploitation of new investigative techniques. This is a consequence of the use of official law enforcement records and the reactivation of previously unresolved cases, and their subsequent entry into the computerized records system introduces a unique factor that could skew the perception of crime occurrence over time, leading to the appearance of disproportionate reporting by year. Specifically, cases originating from periods before the implementation of the digital system are logged as current entries in the system. This process introduces a temporal anomaly in the data, as these reactivated cases inflate the number of recorded incidents in years following the digital system’s adoption.

It is a computerized police database in which all police services in Quebec are obliged to record information from police investigation files (CRPQ). This system records mandatory information on the circumstances of the crime (e.g., location, the weapon used, suspect’s actions, duration of actions, when they began, when they ended, and when he was denounced), on the suspect himself (e.g., age, sex, physical characteristics, place of residence, etc.), on his victim(s) (e.g., age, sex, physical characteristics, place of residence, etc.), the type of relationship between the suspect(s) and the victim(s), the date the investigation began, the date the investigation ended, as well as certain characteristics of the crime under investigation, including, for example, the suspect’s state of intoxication or the use of various types of weapons. To the best of our knowledge, there is no missing data in our dataset, as all cells were completed, with the understanding that this completion was primarily based on the count of the presence of the characteristics, rather than providing a detailed account of their specific nature or context. However, two variables—use of a weapon and alcohol/drug use—could be exceptions, as their logging is not mandatory in the system. For these variables, the choice was made to simply count the presence or absence of the characteristics, operating under the assumption that the data accurately reflects the observations of the investigators. The database extraction included all individuals involved in IPV cases reported to judicial authorities in the province of Quebec from 1966 to 2021. IPV cases were selected using three criteria: (1) the nature of the relationship between the suspect and the victim as part of a case involving a crime against the person (partner or ex-partner, whether those involved were living together or separated, no matter the gender), (2) mention of IPV in the field designed to label a particular feature of the crime under investigation, and (3) explicit mention of the occurrence of IPV in the computer system remark field. The sample from these criteria was comprised of 183,683 individuals involved (i.e., arrested) in at least one incidence of IPV. Analysis of their criminal careers showed that these individuals had been involved in a total of 2,957,379 criminal events (both IPV and other).

For the purposes of this study, we selected only individuals involved in an attempted or completed intimate-partner homicide. An attempted IPH was defined as an event in which an individual survived an injury, caused by a weapon or physical violence alone, that could have led to death. These cases are characterized by (in addition to the victim’s report) evidence of unambiguous intent to kill on the part of a perpetrator who was a current or former intimate partner. Each case was examined by a police officer to validate the presence of intent to kill. A completed homicide is defined as the victim’s death, either at the time of the offense (i.e., at the crime location) or as a consequence of the offense (e.g., at the hospital). A total of 1,780 individuals (0.96% of the sample) were identified as involved in cases of homicide and attempted homicide (unless otherwise stated, the term homicide is used to refer to both attempted and completed crimes) between 1966 and 2021. Cases were distributed as follows: 0.45% (*n* = 8) of cases reported between 1966 and 1980, 4.94% (*n* = 88) reported between 1981 and 1990, 41.80% (*n* = 744) reported between 1991 and 2000, 29.72% (*n* = 529) reported between 2001 and 2010, and 23.10% (*n* = 411) reported between 2010 and 2021. The large variation in the number of homicides by period is due to the involvement of multiple police services in the deployment of the computerized system, which was gradually integrated into it during the 1980-1990 decade. As a result, prior to the early 1990s, the system did not account for all intra-marital homicides occurring within the Canadian province of Quebec.

Perpetrators were predominantly male (84.94%, *n* = 1,512) and middle-aged at the time of the successful or attempted homicide, with an average of 40.96 years of age (*SD* = 12.95). Victims of the successful or attempted homicide were largely female (71.34%, *n* = 1,270) and also around 40 years old, with an average of 39.15 years of age (*SD* = 15.72).

### Measures

#### Main Model

We used a total of 12 continuous variables to examine the career criminal patterns of individuals involved in cases of IPH or attempted homicide. These variables capture the number of offenses perpetrated by an individual during an entire criminal career and are divided into two groups of indicators: those offenses involving an intimate partner and those committed in a non-intimate partner context. The variables related to offenses involving an intimate partner are: (1) number of sexual offenses ( $\bar{x}$ = 0.1; *SD* = 0.3; range 0–3), (2) number of violent offenses ( $\bar{x}$ = 1; *SD* = 2.2; range 0–25), (3) number of failures to comply (e.g., breach of probation conditions) ( $\bar{x}$ = 0.2; *SD* = 0.4; range 0–13), (4) number of other offenses (e.g., Offense against public order, public mischief, hindering the peace officer, etc.) ( $\bar{x}$ = 0.2; *SD* = 0.4; range 0–5), and (5) number of offenses against property ( $\bar{x}$ = 0.2; *SD* = 0.6; range 0–6). The variables related to criminal career in a non-intimate partner context are (6) number of homicides and attempted homicides ( $\bar{x}$ = 0.1; *SD* = 1; range 0–40), (7) number of sexual offenses ( $\bar{x}$ = 0.1; *SD* = 0.5; range 0–10), (8) number of violent offenses ( $\bar{x}$ = 1.1; *SD* = 2.6; range 0–35), (9) number of failures to comply ( $\bar{x}$ = 1.5; *SD* = 4; range 0–50), (10) number of other offenses ( $\bar{x}$ = 0.6; *SD* = 1.5; range 0–21), (11) number of offenses against property ( $\bar{x}$ = 1.2; *SD* = 5.3; range 0–131), and (12) number of drug offenses ( $\bar{x}$ = 0.4; *SD* = 1.4; range 0–17).

#### Criterion Variables

To test the external validity of the main model, we used the following 17 variables related to a perpetrator’s criminal career and the characteristics of the context in which the crime occurred.

##### Criminal career indicators

Six additional continuous variables related to an perpetrator’s criminal career were used: (1) age at first criminal event ( $\bar{x}$ = 37.1; *SD* = 13.3; range 11–89), (2) age at first event of intimate partner violence ( $\bar{x}$ = 38.2; *SD* = 13; range 14–89), (3) length of criminal career in years ( $\bar{x}$ = 6.5; *SD* = 8.1; range 0–38) (i.e., length between first and last offense), (4) total number of criminal incidents ( $\bar{x}$ = 7. 8; *SD* = 14.1; range 1–179), (5) variety of total offenses ( $\bar{x}$ = 3.1; *SD* = 2.4; range 1–11; i.e., Offenses subtype summed in a scale of 1 to 11: homicide, sexual, assault, loss of freedom, uttering threat, property, failure to comply, traffic, fraud, drug, other), and (6) general frequency (i.e., average number of offenses per year; $\bar{x}$ = 0.4; *SD* = 0.8; range 0.02-11).

##### Crime context indicators

A total of nine variables were used to describe the crime context: (7) perpetrator was male (0 = 15.05%; 1 = 84.94%), (8) victim was female (0 = 28.65%; 1 = 71.35%), (9) homicide was completed (as opposed to an attempt; 0 = 67.14%; 1 = 32.86%), (10) alcohol and/or drugs use involved (0 = 86.91% %; 1 = 13.09%), (11) perpetrator and victim were in a relationship at the time of the offense (as opposed to ex-relationship; 0 = 36.29%; 1 = 63.71%), (12) perpetrator used a weapon (0 = 23.82%; 1 = 76.18%), (13) perpetrator and victim where living together at the time of the homicide (0 = 49.43%; 1 = 50.57%), (14) perpetrator’s age at the time of the homicide or attempted homicide ( $\bar{x}$ = 38.97; *SD* = 12.85; range 15–88), and (15) victim age at the time of the homicide or attempted homicide ( $\bar{x}$ 39.15 = 0.4; *SD* = 15.72; range 14–89).

### Analytical Strategy

The analytical strategy followed a two-step process. First, a latent profile analysis (LPA; i.e., including continuous variables) was computed using the Latent Gold V6.0 software package to identify the criminal career patterns (main model variables) of individuals involved in cases of IPH or attempted homicide. LPA is a statistical procedure used to identify heterogeneity that is not directly observable or measurable, making it possible to detect underlying patterns in a set of data or among subgroups of individuals who share important behavioral characteristics (Bray, 2021). The goal of this procedure is to identify mutually exclusive profiles using continuous variables (Lanza et al., 2007). LPA analysis is similar to cluster analysis but provides stronger models as it attributes profile membership mean scores to each individual case. LPA has been used in recent years to identify heterogeneity in the criminal career patterns of different types of perpetrators (Vaughn et al., 2014) and allows a much more in-depth analysis of the criminal trajectories followed by individuals. We utilized the Expectation-Maximization algorithm method to perform LPA on our continuous data. This method, recognized for its capability in effectively estimating profile parameters from continuous data distributions, enabled us to precisely model profiles from observed variables.

Eight models were computed and analyzed from a one-to-eight-profile solution (Table 1). To identify the model with the best fit, multiple indicators are employed, as the traditional likelihood ratio test cannot be used in LPA to guide the decision toward the best model (Masyn, 2013). Thus, the present study used a mix of the Bayesian Information Criterion (BIC), the Vuong-Lo-Mendell-Rubin Adjusted likelihood ratio test (VLMR), and entropy to evaluate the model fit and determine the number of profiles to use in LPA. More specifically, a lower BIC value indicates an improvement in the fit of models (Schwartz, 1978), a significant and lower VLMR value compared with the neighborhood solution indicates a better fit to data, and a higher entropy indicates a better percentage of correct classification (Schwartz, 1978). We tested for multicollinearity and no VIFs were above 2.225, while tolerance values were not below 0.449. The rule of thumb for VIF is typically to be below 4 or 10, depending on the authors (O’brien, 2007). As we utilized data spanning a 55-year time period (1966–2021), it seemed important to test the stability of the initial model. We thus conducted the same LPA using only individuals who initiated their criminal careers after 2000, constituting a subsample of 940 individuals. The results presented in Appendices A and B demonstrate that the five-profile model is the most appropriate, similar to the general model (i.e., the one including all data). Furthermore, the analysis of the five-profile solution concerning the subsample reveals no significant differences from the general model, both in terms of profile content and sample distribution among the different profiles, which follows the same trend.

**Table 1:** Fit Indices for Latent Profiles (*N* = 1,780)

No. of classes	BIC	AIC	Adjusted BIC	L²	VLMR	*p* value	Entropy *R*²
1-Class	24,857.24	24,122.34	24,431.53	9,762.96	-	-	1.00
2-Class	23,895.39	23,105.64	21,861.16	8,038.86	2,613.45	.000	.98
3-Class	22,822.48	21,977.89	21,450.40	6,891.12	453.84	.008	.94
4-Class	22,694.78	21,795.34	21,348.96	6,688.57	144.52	.000	.91
**5-Class**	**22,632.90**	**21,623.78**	**21,246.23**	**6,477.01**	**67.52**	**.003**	**.88**
6-Class	22,633.61	21,679.33	21,270.68	6,552.56	121.35	.000	.85
7- Class	22,677.53	21,613.56	21,256.74	6,446.79	32.57	.003	.85
8- Class	22,710.37	21,591.56	21,252.15	6,404.79	47.66	.000	.81

*Note*. Boldface type indicates the selected model. Vuong-Lo-Mendel-Rubin likelihood ratio test not applicable for the one-class model.

As a second step, we used additional variables to test the external validity of the model and improve its depth. Specifically, this procedure makes it possible to distinguish whether the differences identified in the main classification model and based on a limited number of indicators are reflected in other indicators qualifying the studied phenomenon. To execute this procedure, the data were exported from Latent Gold V6.0 to the Statistical Package for the Social Sciences (SPSS) V.29. During this process, a categorical variable was generated to identify the five profiles in the dataset, reflecting each individual’s most likely profile assignment. Bivariate analyses (i.e., chi-square analysis and Kruskal-Wallis test) were used to identify significant differences between the different profiles. Additionally, post hoc tests (i.e., pairwise Wilcoxon and Z tests) have been used to identify specific differences. A significant global test indicates that there are differences between groups, but it doesn’t specify which ones. Post hoc tests help identify the pairs of groups that differ, providing more detailed information about the nature of these differences. Such a procedure is commonly used with LPA (Vaughn et al., 2014) and makes it possible to distinguish whether the differences identified in the main classification model, based on a limited number of indicators, are also found for other indicators in the phenomenon being studied.

### Ethics

This study received ethical approval from the ethics committee of the University of Montreal (2022–102-D).

## Results

### Latent Profile Analysis

To examine the different criminal career trajectories of individuals involved in IPH or attempted homicide, we used 12 dichotomous variables related to the type of previous criminal incidents. One-to-seven-profile solutions were analyzed to determine the best latent profile solution, with the five-profile solution being the best fit according to the BIC (22,632.90; Table 1 describes the model fit indices for the latent classes). BIC decreased up to Profile 5 but increased from Profile 6 on. A smaller BIC suggests that the trade-off between fit and parsimony has been achieved. The entropy for the five-profile solution was high (0.88), suggesting that the predictors used were appropriate to classify the cases and that classes were highly distinct (Schwartz, 1978). The VLMR indicates that the five-profile model was a significant improvement on the fit of the four-profile model.

**Figure 1:**
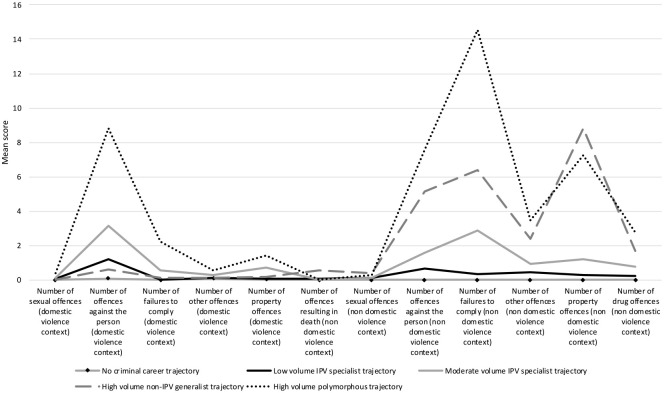
Profile of Five Latent Classes—Mean Score of the Criminal Carrier Based on Class Membership (*N* = 1,780)

Table 2 and Figure 1 show the five-profile solution, with five criminal career patterns for individuals involved in IPH or attempted homicide cases. The largest subgroup is Profile 1 (46.67% of cases, *n* = 813), while the smallest is Profile 5 (3.93% of cases, *n* = 70).

**Table 2:** Profile of Five Latent Classes—Mean Score of the Criminal Carrier Based on Class Membership (*N* = 1780)

	No criminal career trajectory	Low volume IPV specialist trajectory	Moderate volume IPV specialist trajectory	High volume non-IPV generalist trajectory	High volume polymorphous trajectory
*n* =	813	569	194	134	70
Sample %	45.67	31.97	10.90	7.53	3.93
	$\bar{x}$ (*SD*)	$\bar{x}$ (*SD*)	$\bar{x}$ (*SD*)	$\bar{x}$ (*SD*)	$\bar{x}$ (*SD*)
Intimate partner context					
Number of sexual offenses	0.01 (*0.09*)	0.07 (*0.29)*	0.14 (*0.42)*	0.04 (*0.21)*	0.39 (*0.67)*
Number of violent offenses	0.10 (*0.34*)	1.20 (*3.17*)	3.17 (*1.98)*	0.63 (*0.89)*	8.81 (*4.41)*
Number of failures to comply	0.01 (*0.08*)	0.01 (*0.11)*	0.58 (*0.95)*	0.13 (*0.33)*	2.23 (*2.49)*
Number of other offenses	0.09 (*0.31*)	0.15 (*0.42)*	0.32 (*0.61)*	0.11 (*0.32)*	0.59 (*0.97)*
Number of property offenses	0.05 (*0.25*)	0.10 (*0.31)*	0.73 (*0.94)*	0.20 (*0.52)*	1.47 (*1.42)*
Non-intimate partner context					
Number of offenses resulting in death	0.01 (*0.10*)	0.06 (*0.29)*	0.04 (*0.19)*	0.57 (*3.68)*	0.04 (*0.20)*
Number of sexual offenses	0.00 (*0.04*)	0.15 (*0.51)*	0.15 (*0.47)*	0.40 (*1.10)*	0.27 (*0.74)*
Number of violent offenses	0.00 (*0.06*)	0.66 (*1.00)*	1.59 (*1.53)*	5.13 (*3.92)*	7.57 (*5.96)*
Number of failures to comply	0.01 (*0.10*)	0.37 (*0.69)*	2.90 (*2.32)*	6.37 (*5.38)*	14.56 (*8.82)*
Number of other offenses	0.02 (*0.16*)	0.44 (*0.84)*	0.92 (*1.39)*	2.42 (*2.72)*	3.46 (*3.02)*
Number of offenses against property	0.01 (*0.08*)	0.40 (*0.79)*	1.45 (*1.71)*	10.19 (*19.29)*	8.43 (*9.16)*
Number of drug offenses	0.01 (*0.04*)	0.23 (*0.67)*	0.79 (*0.29)*	1.71 (*2.56)*	2.76 (*3.76)*

Profile 1 was labeled as *no criminal career trajectory*. Individuals in this group did not have a criminal career prior to their successful or attempted IPH. Means for all types of offenses studied approximate zero, both for offenses committed in an intimate partner context and those committed in a non-intimate partner context.

Profile 2 is labeled as *low volume IPV specialist trajectory* (31.97% of cases, *n* = 569). Like Profile 1, most of the averages for analyzed offense types were close to zero. Individuals in this profile have an average number of 1.20 violent offenses in an intimate partner context.

Profile 3 is the *moderate volume IPV specialist trajectory* (10.90% of cases, *n* = 194). The criminal careers of these individuals are characterized by an average number of 3.17 violent offenses in an intimate partner context before becoming involved in an IPH or its attempt. The criminal careers of these individuals were also characterized by criminal incidents committed in a non-intimate partner context, with an average number of 1.59 offenses against a person, 2.90 failures to comply, and 1.45 offenses against property in a non-intimate partner context.

Profile 4 is the *high-volume non-IPV generalist trajectory* (7.53% of cases, *n* = 134). Individuals in this profile are characterized by a criminal career in a non-intimate partner context. They presented a high average number of violent offenses, to comply, other offenses, offenses against property, and drug offenses. They also had the highest average number of offenses leading to death or determined to be an attempt to cause death in a non-intimate partner context. These individuals commit on average less than one offense in any type of IPV offense.

Profile 5 is the *high-volume polymorphous trajectory* (3.93% of individuals, *n* = 70). Individuals in this group had a very prolific criminal career, in both an intimate partner and a non-intimate partner context, with an average number of 8.81 offenses against a person, 2.23 failures to comply, and 1.47 offenses against property in an intimate partner context. These individuals also had a high average number of violent offenses, failures to comply, other offenses, offenses against property, and drug offenses in a non-intimate partner context.

### External Validity Analysis

Table 3 presents the findings of an external validity analysis using additional criminal career and crime context indicators. Post hoc tests were conducted to determine significant differences among specific trajectories. The findings revealed significant differences across the five trajectories concerning the number of IPV incidents (ω² = 0.15). Individuals with a higher number of IPV incidents were more likely to be associated with Profile 5. In addition, significant differences were observed in terms of individuals’ age at their first offense overall (ω² = 0.15) and in an IPV context (ω² = 0.15). Individuals who were, on average, younger at the time of their first offense were more likely to be part of Profiles 3, 4, or 5, whereas those with an older average age tended to fall into Profile 1. Similarly, individuals who were, on average, younger at the time of their first offense in an IPV context were more likely to be included in Profile 3, 4, or 5, while those with older average age were more likely to be in profile 1. The indicator for the length of the criminal career also varied among the five profiles (ω² = 0.58). The findings demonstrated that individuals in Profiles 1 and 2 were less likely to have longer criminal careers compared to those in Profiles 3, 4, and 5. Similarly, the total number of criminal incidents in the career (ω² = 0.69), variety of total offenses (ω² = 0.79), and general frequency were associated with the five profiles (ω² = 0.32). Specifically, the results indicated that these three indicators exhibited higher average scores for individuals included in Profile 5 compared with the other profiles. Results demonstrated that perpetrator gender was significantly associated with the five-profile solution (*p* = .003, φ = 0.09). Individuals included in Profile 1 were less likely to be male compared with those included in the other profiles. Furthermore, findings indicated that factors such as homicide completion (φ = 0.28), weapon involvement (φ = 0.20), having a relationship with the victim (φ = 0.09), alcohol and/or drug use (φ = 0.15), and the perpetrator and victim cohabiting at the time of the offense (φ = 0.09) were associated with the five-profile model. Specifically, post hoc tests indicated that the completion of homicide, the presence of a relationship between the perpetrator and victim, cohabitation, or the use of a weapon were more likely to be linked to Profile 1 than to other profiles. Conversely, the use of alcohol/drugs was less likely to be associated with profile 1. Moreover, the results showed that the perpetrator’s age (ω² = 0.06) and the victim’s age (ω² = 0.02) at the time of the homicide exhibited significant differences across the five profiles. Perpetrators and victims with an older average age at the time of the homicide were more likely to be categorized into Profile 1.

**Table 3:** External Validity Analysis of the Criminal Career and Crime Context Correlates for the Five-Profile Membership (*N* = 1,780)

	No criminal career trajectory		Low-volume IPV specialist trajectory		Moderate-volume IPV specialist trajectory		High-volume non-IPV generalist trajectory		High-volume polymorphous trajectory			
*n* =	813		569		194		134		70			
Sample %	45.67		31.97		10.9		7.53		3.93			
	Mean/*n* =	*SD*/%	Mean/*n* =	*SD*/%	Mean/*n* =	*SD*/%	Mean/*n* =	*SD*/%	Mean/*n* =	*SD*/%	Kruskal-Wallis H/X2	ϕ/ω^2^
Criminal career correlates												
Number of IPV incidents	1.30_a_	0.64	2.04_b_	1.22	6.01_c_	2.72	2.75_d_	1.59	14.71_e_	7.08	880.59	0.07
Age at first criminal event	42.24_a_	14.24	35.53_b_	10.77	30.15_c_	9.79	28.20_c_	9.23	27.51_c_	7.59	283.45	0.02
Age at first criminal event of IPV	42.24_a_	14.24	37.07_b_	11.14	31.57_c_	9.84	31.84_c_	9.5	30.17_c_	7.13	190.56	0.01
Length of criminal career	0.00_a_	0	10.30_b_	7.49	12.72_c_	7.02	15.83_d_	6.17	17.03_c,d_	5.21	1,498.20	0.06
Total number of criminal incidents	1.35_a_	0.72	4.35_b_	2.24	13.85_c_	5.03	29.54_d_	21.71	51.79_e_	22.84	1,482.76	0.07
Variety of total offenses (1-11)	1.27_a_	0.59	3.28_b_	1.28	6.03_c_	1.57	6.96_d_	1.57	8.11_e_	1.46	1,421.84	0.08
General Frequency	0.16_a_	0.86	0.22_b_	0.19	0.71_c_	0.46	1.30_d_	0.95	2.12_e_	0.91	1,197.75	0.03
Crime context correlates												
Perpetrator was male	666_a_	81.92	486_a_,_b_	85.41	177_c_	91.24	119_b_,_c_	88.81	64_b_,_c_	91.43	15.78	0.09
Victim was female	575_a_	70.73	405_a_	71.18	143_a_,_b_	73.71	89_a_	66.42	58_b_	82.86	6.82	0.06
Completed homicide	375_a_	46.13	154_b_	27.07	24_c_,_d_	12.37	29_b_,_d_	21.64	3_c_	4.29	143.96	0.28
Alcohol / drugs use	67_a_	8.24	85_b_	14.94	31_b_,_c_	15.98	32_c_	23.88	18_c_	25.71	43.46	0.15
Perpetrator and victim were in relationship at the time of the offense	553_a_	68.02	355_b_	62.39	111_b_	57.22	77_b_	57.46	38_b_	54.29	15.45	0.09
Perpetrator used a weapon	673_a_	82.78	432_b_	75.92	123_c_	63.4	94_b_,_c_	70.15	34_b_,_c_	48.57	69.08	0.20
Perpetrator and victim were living together at the time of the homicide	448_a_	55.1	271_b_	47.63	87_b_	44.85	59_b_	44.03	35_a_,_b_	50	13.50	0.09
Perpetrator was male	42.24_a_	14.24	37.80_b_	11.12	34.06_c_	10.76	33.72_c_	9.35	34.07_c_	8.91	106.87	0.06
Victim age at the time of the homicide	41.27_a_	16.79	37.81_b_	13.9	35.62_c_	15.3	37.84_c_,_b_	15.65	37.79_a_,_b_	15.01	34.30	0.02

Notes. Pairwise comparisons: Each subscript letter denotes a subset whose column proportions do not differ significantly from each other at the 0.05 level. Pairwise Wilcoxon and Z tests have been used.

Omnibus group differences were all significant at *p* < .01 except for female victims (ns).

## Discussion

This study focuses on the criminal careers of IPH perpetrators. We formulated two hypotheses, based on the results of previous studies: (1) IPH perpetrator criminal career trajectories were heterogeneous and (2) specific IPH context characteristics could be associated with these trajectories. To test these hypotheses, we used an LPA with a sample of 1,780 individuals who had been involved in IPH events (attempted or completed) in Quebec. The LPA was performed using a total of 12 indicators that characterized offenses in a domestic violence context to examine the dimensions of participation and frequency in IPH events according to the criminal career paradigm (Blumstein et al., 1986). The results of the analyses validated both hypotheses.

### Evidence of Heterogeneity in IPH Perpetrator Criminal Career and Profile

The results of our study highlight that the criminal career trajectories of IPH perpetrators are heterogeneous. This heterogeneity is apparent for all dimensions of the criminal career paradigm, with a continuum in the involvement, frequency, seriousness, and duration of the different trajectories. These results are new and expand on previous studies (Dixon et al., 2008; Dobash & Dobash, 2016; Vignola-Lévesque & Léveillée, 2021) as well as contradicting studies that suggest that IPH perpetrators were more likely than other individuals who commit homicide to have a criminal history (Dobash et al., 2004; Eriksson et al., 2019; Thomas et al., 2011). This also reflects the evidence found in prior work about IPV in general, individuals participating in this type of criminality are a highly heterogeneous bunch (see Peters et al., 2022). While a majority of IPH perpetrators in our sample had a criminal career, our results suggest that those in Category 1, the largest category, were generally unknown to police. This difference from previous studies could be explained by the Yule-Simpson paradox (Simpson, 1951), a statistical paradox that suggests that a phenomenon observed in multiple groups may not be found when the groups are combined. The pooled analysis of IPH perpetrators’ criminal career information might then suggest that these individuals, considered as a whole, were more likely to be found to have a criminal trajectory because the mean of the indicators for those with such a trajectory “contaminates” the mean for those who do not. This observation illustrates the importance of finding the right compromise between the generalization required by scientific practice and the heterogeneity that characterizes studied populations. However, it is also possible that our use of a larger sample than those in previous studies made it possible to identify phenomena that were more difficult to observe with smaller samples.

Our results also suggest diversity in profiles and contexts. This diversity is particularly important as there were significant differences in all indicators used to test the external validity of the five-profile model. While the general trend from prior studies indicates that IPH generally involves a male perpetrator and a female victim who were in a relationship at the time of the offense (e.g., Eke et al., 2011), our analyses suggest that these characteristics are associated with criminal career trajectories. This observation is also valid for risk factors associated with IPH (i.e., alcohol/drug abuse and use of weapons). It is interesting to note that the heterogeneity of the trajectories identified in this study is consistent with the results of previous studies of IPV perpetrators more generally (see e.g., Holtzworth-Munroe & Stuart, 1994; Peters et al., 2022). This might suggest that there may be general criminal career patterns among IPV perpetrators, with some specificity that could characterize IPH perpetrators.

### Toward a New Criminal Career Trajectory Classification of IPH Perpetrators

The LPA suggested that the best solution to classify the criminal trajectory of IPH perpetrators includes five profiles. From indicators characterizing the number of offenses committed in a domestic and non-domestic context, we identified the following trajectory profiles: *no criminal career, low-volume IPV specialist, moderate-volume IPV specialist, high-volume non-IPV specialist, and high-volume polymorphous* trajectories.

#### No Criminal Career Trajectory

This first profile includes IPH perpetrators who have no official criminal record. This group had been identified in a handful of previous studies (see e.g., Dobash et al., 2009 who labeled this pattern “out of the blue”). Our study is, however, the first to find such a high occurrence of this trajectory — almost 50% – for IPH perpetrators. It is important to remain cautious about the lack of previous crimes for those in this trajectory as the finding is based on official criminal records and could involve individuals whose previous crimes (i.e., whether in a domestic context or not) have not been reported. Interestingly, this trajectory is the one most strongly associated with the use of a weapon and the death of the victim (i.e., completed IPH). We know that the presence of a criminal career is strongly correlated with a low level of self-control, which leads to the more frequent use of physical violence (Gottfredson & Hirschi, 1990). It is possible that individuals without criminal careers are less likely to use physical violence to commit a violent assault or homicide but more likely to use a weapon, which increases the risk of death for the victim. This trajectory might include those in the *mentally ill* category proposed by Kivisto (2015), which includes individuals with various mental disorders who had not committed IPV prior to the homicide. However, this suggestion could be nuanced as some studies have found that mentally ill persons are more likely to have a criminal career than those who are not (Wallace et al., 2004). Some of these homicides might be the result of attacks for instrumental purposes (similar to those in the *low criminality and low psychopathology* category of Dixon et al., 2008) or as a final strategy to avoid the partner leaving (see the *unstable dependent partner* category of Vignola-Lévesque & Léveillée, 2021). Finally, it is interesting to observe that this trajectory includes the largest number of women, who might be more likely to use a weapon to defend themselves or to dominate in a physical confrontation with a man.

#### Low Volume IPV Specialist Trajectory

This criminal career trajectory is defined by a shorter history of non-lethal domestic violence, with its incidence limited or at least unknown to authorities. This trajectory agrees with the *undercontrolled/dysregulated* category proposed by Kivisto (2015), which includes individuals involved in episodic IPV, particularly those who exhibit borderline personality disorders. However, Kivisto’s (2015) also suggests that IPH perpetrators in this category have high levels of substance use, which is not supported by our results. This discrepancy could be explained by the fact that Kivisto’s typology is based on samples from a forensic psychiatric context, which increases the likelihood that it will include individuals with psychological disorders or problems with substance abuse. While the data we used included a much broader spectrum of profiles and is therefore more likely to be reliable, it is also possible that information related to alcohol/drug use was less accurately recorded. Finally, we found that the average age at the first IPV event and at the time of the IPH are similar. Such a result could suggest that escalation in violence occurs quickly, and authorities have little time to intervene to prevent its occurrence.

#### Moderate-Volume IPV Specialist Trajectory

This profile is characterized by the presence of a more extensive and longer IPV criminal career. Although it is primarily characterized by an increase in IPV over time, our results suggest that the criminal careers of those in this category begin with acts of violence committed outside the domestic context. This criminal career trajectory agrees with the *controlling violence partner* category (Vignola-Lévesque & Léveillée, 2021) and the *moderate-high criminality and high psychopathology* category (Dixon et al., 2008) as well as the *undercontrolled/dysregulated* category proposed by Kivisto (2015). All these categories include criminal pathways into moderate violence in domestic and non-domestic settings. Furthermore, these studies highlight that IPH perpetrators in these categories are distinguished by feelings of anxiety and jealousy (Dixon et al., 2008; Kivisto, 2015).

#### High-Volume Non-IPV Specialist Trajectory

Individuals included in this profile show a singular trajectory. In one sense, they are similar to those in the no criminal career trajectory as they have a low probability of a history of IPV. However, they reveal an extensive criminal career outside the context of domestic violence, with offenses related to interpersonal assault, property violation, and drug-related offenses. This category is congruent with the *high criminality and low-moderate psychopathology* category proposed by Dixon et al. (2008) as well as the *mentally ill* (Kivisto, 2015) and *controlling violence partner* categories (Vignola-Lévesque & Léveillée, 2021). Individuals with this criminal trajectory most probably exhibit an antisocial personality profile characterized by a lack of legal employment and a high level of involvement in a variety of criminal activities ranging from interpersonal violence to drug use (Dixon et al., 2008).

#### High-Volume Polymorphous Trajectory

This trajectory, the least prevalent, is characterized by a particularly prolific criminal career that includes both domestic and non-domestic crimes and involves the longest duration and greatest variety and frequency of criminal acts. This criminal trajectory could correspond to Kivisto’s (2015) *chronic batterer* category as well as Vignola-Lévesque & Léveillée’s (2021) *generally angry/aggressive partner* category. Individuals with these criminal trajectories are characterized by antisocial, sadistic, and narcissistic personality disorders (Kivisto, 2015). The homicide is part of a general pattern of violence, possibly the result of a violent event going too far. However, it is interesting to note that, despite the extreme profile of individuals in this category, their rate of completed homicide is the lowest. Two non-mutually exclusive hypotheses can be proposed to explain this. The first is that individuals who follow this trajectory also have the highest rate of alcohol/drug use and as suggested in previous studies, alcohol/drug abuse weakens and disorients perpetrators, making a lethal outcome less likely (see e.g., Overstreet et al., 2021). Second, IPH perpetrators in the high-volume polymorphous trajectory are also the least likely to use a weapon. Several studies have shown that using only physical violence reduces the likelihood of lethal outcomes in IPV (Campbell et al., 2017; Cooper & Smith, 2011; Overstreet et al., 2021; Spencer & Stith, 2020).

## Conclusion

This study is the first to explore the criminal career trajectories of IPH perpetrators and its findings have both theoretical and practical implications. The results suggest that there is a high degree of heterogeneity in the criminal trajectories of individuals involved in IPH, which reflecting general IPV knowledge about population heterogeneity, as well as in the contextual characteristics of these criminal events among IPH.

This research is not without limitations. First, official police data do not provide a complete picture of the occurrence of IPV, which is still underreported. However, such underreporting is very low when it involves homicide or attempted homicide (Aebi & Linde, 2012). Second, the methodological choice to include both attempted and completed homicides may have influenced the classification of criminal trajectories. However, several previous studies have suggested that the difference between completed and attempted homicide is often explained by situational factors (see e.g., Mieczkowski & Beauregard, 2010). Third, proper class assignment is not guaranteed with latent analysis, which could create classes that do not exist. However, external validity step analysis is used to mitigate this limitation (Weller et al., 2020). Fourth, we used data on a nearly 70-year period. This could result in the most recent cases being over-represented compared with the oldest cases, which were introduced retrospectively. We cannot exclude that some characteristics may be affected by the temporal aspect. Fifth, an inherent limitation of the criminal career concept is that only crimes reported to the authorities are used in characterizing criminal trajectories. Given the high rate of failure to report IPV, we cannot exclude the possibility that our data does not include individuals with no criminal career who have nevertheless committed homicide or attempted homicide. Sixth, another limitation of our study lies in the potential conflation between age and the duration of the criminal career. Finally, we recognize the limitations inherent in our approach, primarily stemming from our reliance on criminal history data. These data, while accessible and informative regarding behavioral patterns, lack the depth provided by psychological variables, which capture the nuances of individual behavior, cognition, environment, motives, and relationships. Such psychological factors have substantial explanatory and theoretical value for understanding IPH.

**Figure 2:**
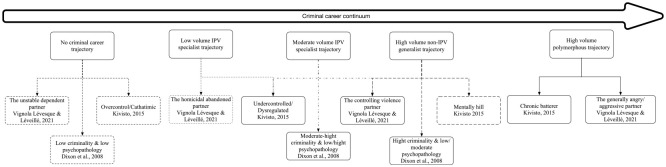
Comparison Between Criminal Trajectories and Previous Empirical/Clinical Classifications of IPH Perpetrators

With regard to theoretical implications, our study shows that the criminal career paradigm is useful in understanding IPH. First, our results demonstrate that there is no single criminal trajectory predictive of IPH. The criminal careers of those implicated in such homicides or attempted homicides show varying combinations of duration, participation, seriousness, and frequency and appear to be on a continuum from no criminal career to a polymorphic criminal career. This suggests that the study of criminal trajectories needs to be combined with the study of other risk factors, whether individual or situational. Second, our results show that IPH has a much higher rate of lethality for individuals without a previous criminal conviction, reinforcing the hypothesis that the explanation of the IPH phenomenon requires the use of several theoretical models. Finally, the empirical typology proposed in this study extends previous studies’ emphasis on psychological factors. Figure 2 shows the interaction of the criminal trajectories identified in our study with categories from previous studies. It is interesting to note that all the trajectories identified in the present study can be associated with previously identified categories.

As to the practical implications, first, the available data suggests that identifying individuals with the highest risk of committing IPH will require the development of assessment tools that include indicators related to both criminal trajectories and individual and situational risk factors. Second, the results reveal that a significant proportion of IPH cannot be predicted solely based on a perpetrator’s criminal trajectory, as a large percentage (e.g., profile 1) virtually do not have one. This finding, suggests that it is important to develop prevention strategies that raise public awareness that IPH does not always occur as part of a criminal trajectory involving violence but may be related to other factors, such as the availability of firearms. Finally, this study provides support for previous findings that IPH perpetrators are not a homogeneous population and that intervention programs tailored to the specific needs of everyone, rather than the “one-size fits all” approach, should be developed.

Future studies should focus on two main research areas. First, further investigation of IPH perpetrators without a criminal career could help understand the underlying motivations that led them to commit homicide, making it possible to develop better ways to prevent lethal domestic violence. Second, to better understand the dynamics involved in IPH, it seems essential to understand what differentiates homicide attempts that end before the death of the victim from those that lead to homicide. Future studies should strive to integrate a multidisciplinary approach, combining criminal history with psychological assessments, to foster a more comprehensive understanding of IPH, thereby enhancing both theoretical insights and practical interventions.

## Supplemental Material

sj-docx-1-cjb-10.1177_00938548241257604 – Supplemental material for One Size Doesn’t Fit All: An Exploratory Typological Approach to Understanding Criminal Career Heterogeneity in Intimate Partner Homicide Supplemental material, sj-docx-1-cjb-10.1177_00938548241257604 for One Size Doesn’t Fit All: An Exploratory Typological Approach to Understanding Criminal Career Heterogeneity in Intimate Partner Homicide by Olivier Péloquin, Julien Chopin, Francis Fortin, Jean-Pierre Guay, Eric Chartrand and Sarah Paquette in Criminal Justice and Behavior
