# Supplementary material for: One Size Doesn’t Fit All: An Exploratory Typological Approach to Understanding Criminal Career Heterogeneity in Intimate Partner Homicide
Source: Crim Justice Behav. 2024 Jun 25;51(11):1734–54. doi: 10.1177/00938548241257604 (PMC11977829; doi:10.1177/00938548241257604)
Supplement: sj-docx-1-cjb-10.1177_00938548241257604 – Supplemental material for One Size Doesn’t Fit All: An Exploratory Typological Approach to Understanding Criminal Career Heterogeneity in Intimate Partner Homicide [file sj-docx-1-cjb-10.1177_00938548241257604.docx]

**Online Supplemental Material**

Appendix 1. Table 1. Fit indices for latent profiles of individuals involved in cases that occurred since 2000 (*n* = 940).

| **Nb of classes** | **BIC** | **AIC** | **Adjusted BIC** | **L²** | **VLMR** | **p-value** | **Entropy R²** |
| --- | --- | --- | --- | --- | --- | --- | --- |
| 1-Class | 209772.40 | 202261.29 | 203811.29 | 108504.02 | - | - | 1 |
| 2-Class | 190262.19 | 182121.11 | 183801.11 | 88103.83 | 20400.18 | 0.002 | 0.88 |
| 3-Class | 186028.84 | 177257.80 | 179067.80 | 82980.53 | 5123.30 | <.001 | 0.85 |
| 4-Class | 185457.96 | 176056.95 | 177996.95 | 81519.68 | 1460.84 | <.001 | 0.83 |
| **5-Class** | **185311.83** | **175280.86** | **177350.86** | **80483.59** | **1036.08** | **<.001** | **0.80** |
| 6-Class | 185329.44 | 174668.51 | 176868.51 | 79611.23 | 872.35 | 0.005 | 0.77 |
| 7- Class | 185753.63 | 174462.73 | 176792.73 | 79145.46 | 465.77 | 0.010 | 0.77 |
| 8- Class | 186297.76 | 174376.90 | 176836.90 | 78799.63 | 345.83 | <.001 | 0.76 |

Note: Boldface type indicates the best model

Vuong-Lo-Mendel-Rubin likelihood ratio test not applicable for one-class model

Appendix 2. Profile of five latent classes of individuals involved in cases that occurred since 2000 - Mean score of the criminal carrier based on class membership - (N=940).

|  | No criminal career trajectory | Low volume IPV specialist trajectory | Moderate volume IPV specialist trajectory | High volume non-IPV generalist trajectory | High volume polymorphous trajectory |
| --- | --- | --- | --- | --- | --- |
| *n* = | 332 | 314 | 143 | 97 | 54 |
| Sample % | 35.32 | 33.40 | 15.21 | 10.32 | 5.75 |
|  | x̄ (*SD*) | x̄ (*SD*) | x̄ (*SD*) | x̄ (*SD*) | x̄ (*SD*) |
| **Intimate partner context** |  |  |  |  |  |
| Number of sexual offences | 0.00 (*0.00*) | 0.04 (*0.22)* | 0.13 (*0.37)* | 0.03 (*0.17)* | 0.43 (*0.72)* |
| Number of violent offences | 0.09 (*0.29*) | 0.74 (*0.93*) | 2.94 (*1.89)* | 1.43 (*1.44)* | 9.04 (*4.56)* |
| Number of failures to comply | 0.01 (*0.11*) | 0.01 (*0.08)* | 0.60 (*0.92)* | 0.15 (*0.36)* | 2.63 (*2.61)* |
| Number of other offences | 0.05 (*0.23*) | 0.17 (*0.44)* | 0.30 (*0.62)* | 0.13 (*0.34)* | 0.76 (*0.97)* |
| Number of property offences | 0.06 (*0.25*) | 0.12 (*0.35)* | 0.76 (*0.97)* | 0.21 (*0.54)* | 1.50 (*1.48)* |
| **Non-intimate partner context** |  |  |  |  |  |
| Number of offences resulting in death | 0.01 (*0.11*) | 0.03 (*0.18)* | 0.02 (*0.14)* | 0.15 (*0.58)* | 0.04 (*0.19)* |
| Number of sexual offences | 0.00 (*0.06*) | 0.11 (*0.41)* | 0.16 (*0.51)* | 0.41 (*1.20)* | 0.31 (*0.82)* |
| Number of violent offences | 0.01 (*0.10*) | 0.64 (*0.95)* | 1.49 (*1.38)* | 5.19 (*3.91)* | 6.39 (*7.54)* |
| Number of failures to comply | 0.02 (*0.12*) | 0.48 (*0.77)* | 3.05 (*2.30)* | 6.37 (*5.51)* | 14.26 (*9.09)* |
| Number of other offences | 0.02 (*0.18*) | 0.50 (*0.89)* | 0.80 (*1.18)* | 3.01 (*3.43)* | 3.12 (*0.76)* |
| Number of offenses against property | 0.01 (*0.95*) | 0.36 (*0.80)* | 1.17 (*1.33)* | 10.20 (*17.51)* | 6.80 (*8.14)* |
| Number of drug offences | 0.00 (*0.00*) | 0.26 (*0.70)* | 0.84 (*1.42)* | 1.88 (*2.65)* | 2.87 (*3.97)* |
